# Epidemiological Aspects of Equid Herpesvirus-Associated Myeloencephalopathy (EHM) Outbreaks

**DOI:** 10.3390/v14112576

**Published:** 2022-11-21

**Authors:** Eva Klouth, Yury Zablotski, Jessica L. Petersen, Marco de Bruijn, Gittan Gröndahl, Susanne Müller, Lutz S. Goehring

**Affiliations:** 1 Center for Clinical Veterinary Medicine, Faculty of Veterinary Medicine, Ludwig-Maximilians University, 80539 Munich, Germany; 2 Department of Animal Science, University of Nebraska, Lincoln, NE 68588, USA; 3 Wolvega Equine Hospital, 8474 EA Oldeholtpade, The Netherlands; 4 National Veterinary Institute (SVE), 75189 Uppsala, Sweden; 5 Baden-Wuerttemberg Animal Health Services, 70736 Fellbach, Germany; 6 M.H. Gluck Equine Research Center, College of Agriculture, Food and the Environment, University of Kentucky, Lexington, KY 40506, USA

**Keywords:** risk factor, boarding facility, age, breed, sex, vaccination

## Abstract

Equid Herpesvirus Myeloencephalopathy (EHM) is a multifactorial disease following an EHV-1 infection in Equidae. We investigated a total of 589 horses on 13 premises in Europe in search of risk factors for the development of EHM. We found that fever (*p* < 0.001), increasing age (*p* = 0.032), and female sex (*p* = 0.042) were risk factors for EHM in a logistic mixed model. Some breeds had a decreased risk to develop EHM compared to others (Shetland and Welsh ponies; *p* = 0.017; *p* = 0.031), and fewer EHV-1-vaccinated horses were affected by EHM compared to unvaccinated horses (*p* = 0.02). Data evaluation was complex due to high variability between outbreaks with regards to construction and environment; viral characteristics and the virus’s transmissibility were affected by operational management. This study confirms earlier suspected host-specific risk factors, and our data support the benefit of high vaccine coverage at high-traffic boarding facilities.

## 1. Introduction

Equid herpesvirus type 1 (EHV-1) is a worldwide cause of respiratory disease in horses; however, infection is also associated with complications, including abortion and Equid herpesvirus-associated myeloencephalopathy (EHM). EHV-1 is a highly contagious disease that spreads horizontally via direct nose-to-nose contact or indirectly via airspace or fomites [1]. Following upper respiratory tract infection, the virus progresses into local lymphatic tissue, and cell-associated viremia in lymphocytes and monocytes may follow [2]. Viremia allows EHV-1 to reach the (small) vasculature of the pregnant uterus and/or CNS, resulting in mononuclear cell extravasation, vasculitis, thrombosis, and ischemia [3]. During EHM, the entire spinal cord can become affected, which will result in impairment of a horse’s gait, causing ataxia, weakness, paralysis, and/or dysmetria [4]. Typically, only the viremic phase in the adult horse is accompanied by fevers (>38.4 °C), whereas the initial respiratory tract infection prior to viremia in the adult is usually not [5,6]. In a stable building or unit with clinical cases of fever and/or EHM, seroconversion has been detected in up to 90% of all occupants, including asymptomatic cases [7]. Of those with fever, only a proportion will develop clinical EHM [8]. Outbreaks can be contained successfully to a single stable unit [9]. 

Whether viremia occurs or differs in magnitude and duration depends on characteristics of the viral strain, cumulative infectious dose, and on the host’s preinfection immunity [4]. EHV-1 infection experiments have shown that some vaccines are able to fully or partially suppress viremia in vaccinated horses compared to unvaccinated controls [10]. Furthermore, a virus-specific factor, a single nucleotide polymorphism (SNP) results in a D-(histidine)- over N (arginine)-amino acid switch in the polymerase gene (ORF30) and likely in more robust shedding and viremia of the infected [11]. 

EHM has been more frequently encountered in some breeds over others. ‘Tall horse’ premises (housing draft horse breeds, Thoroughbreds, Standardbreds, Warmblood breeds, American Paint/Quarter Horses) appear more commonly affected by EHM, whereas significant differences were found on mixed operations co-stabling Shetland/ Welsh ponies. A recently described outbreak in a mixed-breed operation showed significant differences in EHM frequencies between Fjord/Warmblood and Shetland/Welsh ponies [12]. EHM is more frequently reported in the adult horse compared to the (weaned) foal or yearling, with some who believe EHM frequency and severity increases with age [13]. Inconsistently, female horses have been associated with an increased risk for EHM upon infection or with increased disease severity [9,14]. Having had an infection within the past 12 months has also been shown to limit viremia [15]. Two outbreak investigations have suggested an overrepresentation of EHM cases among the EHV-1-vaccinated (or ‘more recently’ vaccinated) over the unvaccinated [14,16]. This discussion is sparked by preliminary reports of an EHM outbreak at a multiple week competitive event in Valencia, Spain in 2021 with similar findings [17]. 

This study takes a retrospective look at nearly 600 horses/ponies of various breeds, stabled at 13 premises with EHV-1 outbreaks with EHM in Germany, Denmark, and Sweden between 2009 and 2020. The aim of the study was to perform a risk factor analysis for occurrence of EHM upon infection with EHV-1. Although similar studies have been conducted on unvaccinated herds in the past, this study includes both EHV-1-vaccinated and unvaccinated animals.

## 2. Materials and Methods

Farm owners/managers, referring veterinarians, or horse owners who called for advice on EHV-1 outbreak management were referred to one of the authors. All premises enrolled were investigated after quarantine had been lifted. We asked farm management and/or DVMs to file a survey, which was then discussed during site visits by the authors (EK, GG, SM). At that time, individual questionnaires were handed out to horse owners. Thirteen outbreaks with 589 horses on different premises in Germany (9), Denmark (2), and Sweden (2) were investigated between 2018 and 2020. We included data from 2 outbreaks already published in 2013 and 2021 [12,18]. Results from owner-filled questionnaires were cross-referenced with farm management or DVM on-site information. The farm owner/management version focused on general information about the farm (region and country; type of operation; type of husbandry; number of horses; breed distribution; number of EHV-vaccinated horses; and layout of the farm with approximate size); specific information about the outbreak (starting date, index case, season, and weather); number of horses with fever and/or EHM; number of euthanized horses; results of postmortem exams; infectious agent and co-infections (strain variant and concurrent infections with other pathogens); the timing and duration of any interventions, quarantine implemented, testing, further laboratory diagnostics, whether horses stayed on the premises or were transported to a hospital or a different farm; and whether other premises in the vicinity were affected, and if so, whether these were in-contact facilities, and what criteria determined lifting quarantine.

The following data were collected from horse owners by questionnaire: (1) animal ID; (2) sex (female, male intact, or male castrated); (3) breed; (4) age at time of outbreak; (5) vaccinated against EHV-1 (or -1/-4); (6) location during the outbreak; (7) was EHV-1 diagnosed in your horse?; (7a) date of first clinical signs; (7b) which clinical signs (none, fever, cough, nasal discharge, (limb) edema, neurological clinical signs (paresis, paralysis, ataxia, tail hypotonia/atonia, urinary incontinence/dysuria, recumbency), other–which?); (7c) treatments; (8) did your horse survive the outbreak? If not, when (date) was euthanasia/death? (9) Anything special you want to mention about the animal? After a few weeks, the questionnaires were collected, and data were sorted and tabulated.

Animals (n = 589) were stratified according to breed, age, and sex (‘male’ or ‘female’). Vaccination for EHV-1/-4 was either ‘yes = 1’ or ‘no = 0’, and 1 required the following criteria: i) primary course of immunization with a licensed product and ii) followed by booster vaccinations at least every 6 months regardless of the follow-up product. Animals that had received vaccines in the past but were not currently vaccinated were categorized 0. Cutoff for ‘fever’ was a rectal temperature ≥38.3 °C, with 0 = no fever and 1 = fever. Clinical signs of neurologic disease, attributable to EHM, including dysuria, were coded 0 (=none) or 1 (=present). We encountered a large diversity of breeds (see Table 1). Warmblood horses (from various studbooks throughout Europe) were the predominant breed with 296 animals, followed by smaller numbers of other breeds. Those with >15 members were the following: American Quarter Horse (n = 46), (Norwegian) Fjord Horse (n = 32), Shetland pony (n = 24), Welsh (A-C) pony (n = 23), and Arabian (n = 16), (Tyrolean) Haflinger horse (n = 16). To include as many animals as possible, we assigned remaining breeds to one of 6 clusters based on genetic background using the information by Petersen et al. (2013) [19] and Felicetti et al. (2010) [20]. The Petersen study shows genetical proximity between 38 defined breeds based on majority rule and neighbor-joining tree calculations, with data acquired from 10,000+ single nucleotide polymorphisms (SNP). Felicetti et al. evaluated similarity in 15 breeds with partial inclusion and overlap with breeds included by Petersen [19]; however, their group allocation was based on microsatellite data. Based on further recommendations (JLP), we added some yet-unassigned breeds to the following clusters: Criollo–cluster 3; Friesian and Haflinger—cluster 4; Connemara and Welsh pony—cluster 5 (British Isles pony breeds), and Konik, Huzul—cluster 6 (Nordic pony, coldblooded breeds). We did not reach consensus for the following breeds: Lipizzaner (n = 1), Lewitz horse (n = 1), Duelmener (n = 1), and Pinto (n = 1). These animals remained unassigned and were excluded from any ‘breed’ calculations. The product of direct (F1) crossbreeding within a cluster resulted in addition of the data to the same cluster (e.g., a Warmblood-Standardbred cross product, 2 horses). Products of crossbreeding between 2 clusters were excluded from the breed analysis (n = 15, Arabian-WB). A sizeable number of animals (n = 67) had no pedigree information and were excluded from ‘breed’ analysis. 

**Figure 1:**
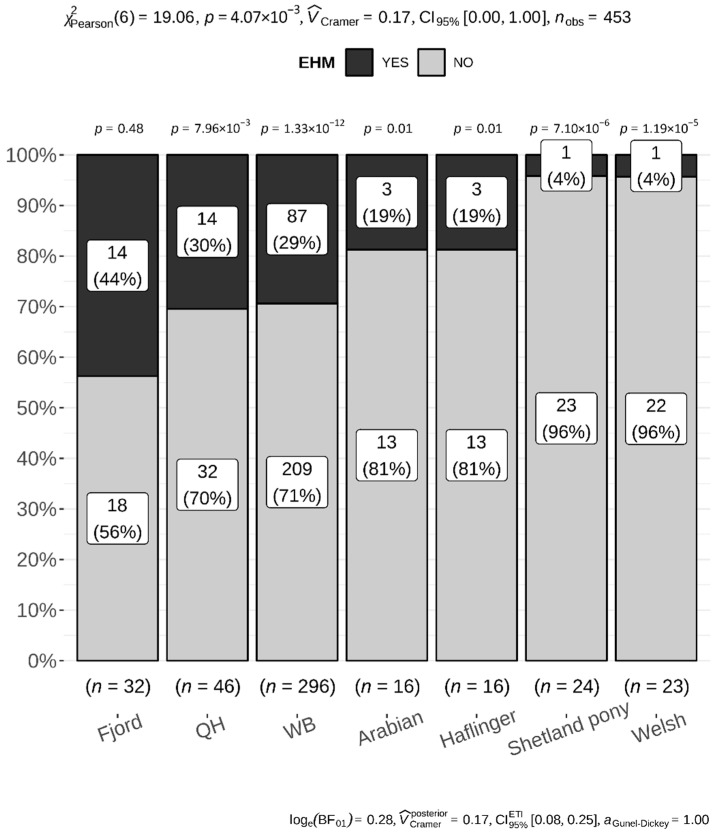
From left to right, 7 distinct breeds (Fjord, Quarter Horse (QH), Warmblood (WB), Arabian, Haflinger, Shetland and Welsh pony) are arranged by decreasing EHM frequency (numbers and percentage as stacked bar plot). The *p*-values above individual stacked bars compare the proportions of EHM-affected (dark gray) individuals to unaffected (light gray) animals within each breed. A chi-square test (top of the plot) finds a significant relationship between breed and EHM (*p* = 0.004), suggesting the need of post hoc pairwise comparisons between individual breeds via Fisher’s tests (reason: low numbers per category). The effect of this relationship is small (V_Cramer_ = 0.17 [21]). In contrast to the *p*-value, the Bayes Factor (BF) provides weak evidence for a relationship between EHM and breed (log(BF01) = 0.28 [22]), whereas the Bayesian effect size suggests a moderately strong relationship (V_Cramer_ = 0.21; 95% Highest Density Intervals 0.22; 0.73). Such disagreement between the frequentist (plot top) and Bayesian methods (plot bottom) might be caused by a low number of observations in several breeds and suggests the need for more data.

**Figure 2:**
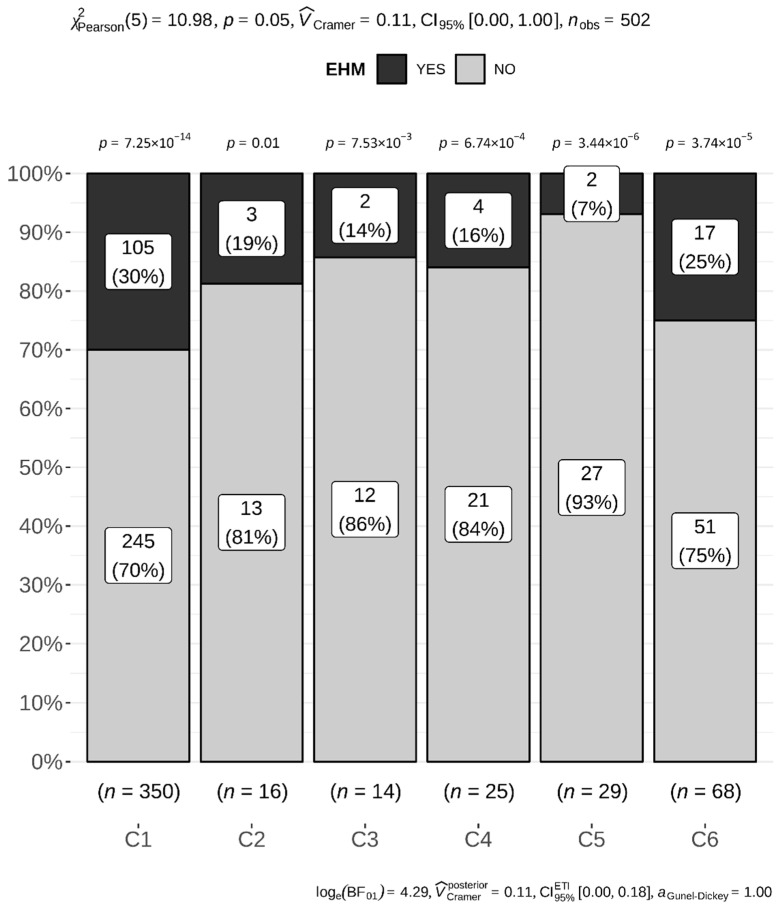
Six breed clusters (1–6) according to similar genetical background are arranged from left to right. EHM frequencies per cluster are shown as percentage in a stacked bar plot (dark grey). The *p*-values above distinct stacked bars compare the proportions of EHM-affected (dark gray) individuals versus unaffected (light gray) animals inside of every particular breed cluster. The chi-square test finds only a suggestive relationship between breed clusters and EHM (*p* = 0.05, low evidence against the null hypothesis). Also, the effect of this relationship is small (V_Cramer_ = 0.11 [21]). The Bayes Factor (BF) supports this result by indicating a very strong evidence for the null hypothesis that there is no relationship between EHM and breed (log(BF01) = 4.29 [22]). The Bayesian effect size also supports this conclusion with a small effect size (V_Cramer_ = 0.15; 95% Highest Density Intervals 0.09; 0.20).

**Figure 3:**
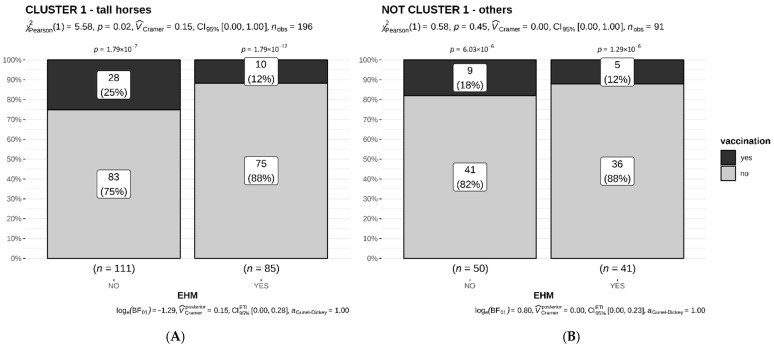
(**A**) Cluster 1 horses (genetically similar ‘tall’ horses) and 3 (**B**): all others (*not*-cluster-1) stratified into animals with EHM (right bar, panel (**A**) or (**B**)), or without EHM (left bar, panel (**A**) or (**B**)), and further stratified vertically into EHV-unvaccinated (light gray) or EHV-vaccinated (dark gray) animals. The *p*-values above individual bars compare the proportions of vaccinated versus unvaccinated animals, indicating fewer animals were vaccinated. The chi-square test (results at top of figure) finds a benefit of vaccination in the prevention of EHM (*p* = 0.02) for cluster 1 horses (panel (**A**)). Those without EHM (left bar) were more frequently (25%) fully vaccinated when compared to horses with EHM, where only 12% were fully vaccinated. The difference in percentages is small (V_Cramer_ = 0.15 [21]). The Bayes Factor (BF) supports this result by indicating a substantial evidence for the benefit of vaccination in EHM prevention (log(BF01) = −1.29 [22]). The Bayesian effect size is, however, also small (V_Cramer_ = 0.16; 95%; Highest Density Intervals 0.03; 0.28). Findings for *not*-cluster-1 animal results were not significant, likely due to small sample size. The chi-square test (results: top of figure, panel (**B**)) finds no relationship between vaccination and EHM (*p* = 0.45, no evidence against the null hypothesis). The difference in percentages is small (V_Cramer_ = 0.0 [21]). The Bayes Factor (BF) supports the latter result by indicating no evidence for the null or for the alternative hypothesis (log(BF01) = 0.80 [22]) and by showing a very small Bayesian effect size (V_Cramer_ = 0.09; 95% Highest Density Intervals 0; 0.23).

Premises A–H and L were in Germany; farms I and M in Denmark; and farms J and K in Sweden. The total number of units and those affected by EHV-1 are listed in Table 2. A unit was considered ‘affected’ based upon the following findings: (i) diagnostic samples positive for EHV-1; (ii) fever; and (iii) EHM. We assumed > 90% of horses within an affected unit were exposed to EHV-1 and (sub)clinically infected [7]. For the ease of calculations, we assumed all animals exposed and infected. We used R 3.6.3 (29 February 2020) statistical software. All variables—breed, age, sex, and vaccination status—were treated as categorical; age was treated as continuous. We fitted a logistic mixed model to predict EHM, with age, sex, vaccination, and fever as fixed effects, and with farm as a random effect. Due to 311 missing out of 3540 observations, a total of 339 animals with complete records were included in this analysis. The proportion of EHM within 7 breeds with >15 members (n = 453) was calculated using a chi-square test, with a proportion test for EHM vs not-EHM for each breed (Figure 1), and a pairwise Fisher’s exact test, with Holm adjustment for multiple comparisons for pairwise comparisons between the breeds. The same methods were applied to analyze EHM proportions in clusters 1–6 (n = 502) (Figure 2). A chi-square test was used to investigate the effect of EHV vaccination on the proportion of animals with EHM. Separate calculations were done for cluster 1 (n = 196) or not-clusters 1 (n = 91) animals (Figure 3). The level of significance was set at α < 0.05. 

## 3. Results

### 3.1. Operation Characteristics

Premises differed in many aspects, including the type of husbandry (individual boxes, paddock boxes, or group husbandry); number and size of units (Table 2); and if they were a mixed-breed vs. single- or predominate-breed operation. All farms allowed boarding to some degree, defined as renting boxes to individual horse owners or accepting horses from elsewhere for a limited time. Some farms had additional functions (breeding (B and C); old horse retirement (E); and riding instruction (E, J, and L). It is noteworthy that the consequence of ‘additional function’ is that a variable proportion of animals on a farm are owned by a single person, often the farm owner/management. The predominant breed on farms A and B was the American Quarter Horse, and Warmblood on farms C, D, F, H, and J–M. In addition to Warmbloods, farms J, K, and M housed many sport ponies without specified breed backgrounds. Farms E, G, and I were mixed-breed operations. 

Most farms (A–D, F, H, J, L, and M) had open sidings between boxes (exception: farm K). Farms A, C, and D had mostly single box aisles; other animals were kept in small group husbandry systems. Farm E had an indoor group husbandry system with groups of 4 to 24 horses. While group-housed during nights, all animals were combined in a large outdoor paddock during the daytime (detailed information: [12]). 

### 3.2. EHM Outbreak Characteristics

Only horses from affected stable units were included in the study. Data on outbreaks in C and E were published previously [12,18]. Unfortunately, a few data sets of affected units were incomplete when filed or not filed at all (Table 2). 

Most outbreaks started between November and April, with the exceptions of farm B (June) and D (July). The outbreaks started differently. On farms A, B, F, G, and K–M, they began with febrile horses and coughing; on farms D, E, H–J with ataxia or recumbency; or with an abortion (farm C). Numbers of febrile horses and EHM cases varied between outbreaks (Table 2). Reported additional clinical signs during an outbreak included abortions (8; farms C and M), distal limb edema (20; farms G, F and I), and coughing (6; farms B, F, J, and L). Polymerase gene polymorphism (N- or D-variant) was determined in 8 outbreaks (Table 2). Among those was outbreak L, with a confirmed N-strain variant. Whereas every third horse was affected by a fever, only one animal developed EHM. 

All farms announced quarantine as soon as an EHV-1 diagnosis was confirmed, with a few starting quarantines upon suspicion while awaiting laboratory confirmation. Treatment and mitigation strategies and the timing of implementation varied highly. The duration of quarantine ranged from 3 to 15 weeks. Typically, quarantine was lifted four weeks after the last horse with fever or neurologic disease was identified. Farm E maintained their daily routine of daytime turnout (exception: recumbent horses, detailed information [12]); also farms J and K turned out non-EHM affected horses during the daytime. Any further information on outbreak propagation and mitigation strategies was mostly unavailable.

### 3.3. Animal Data and Risk Assessment

We included 589 horses/ponies in this study (Table 1). Only those stabled in units with at least one confirmed EHM case, or with a fever attributed to EHV-1 infection were included. EHM incidence in affected units varied (range: 2.2–78.3%; mean: 20.9; SD: 23.0; median: 10.1). 

Comparing all outbreaks in a mixed effects logistic regression, EHM was significantly associated with increasing age, female sex, and fever. The model’s total explanatory power is substantial, with a conditional R^2^ = 0.58, whereas the part of the calculation that relates to the fixed effects alone was 0.25 (marginal R^2^). Age of the horses ranged from 1 to 33 years (mean: 14 years; SD: 6.8 years; median: 13 years; IQR: 9 years). An odds ratio (OR) for age was 1.06 (CI = 1.01–1.12; *p* = 0.032). Treated as a continuous variable, an age increase by one year increased the risk for EHM by 6%. There were 287 male (incl. 3 intact males) and 251 female animals in affected units. Female horses were at greater risk to develop EHM (OR = 1.94; CI = 1.02–3.67; *p* = 0.042). A potent indicator of whether an animal would develop EHM during an outbreak was fever (OR = 13.95; CI = 5.75–33.81; *p* ≤ 0.001) (Table 3). 

We compared 453 horses/ponies of 7 distinct breeds with group sizes > 15 and their respective EHM frequencies. Figure 1 shows the proportions of EHM-affected animals within a distinct breed. Comparing these breeds using pairwise Fisher’s exact test with Holm adjustment for multiple comparisons, Fjord horses and Shetland ponies (*p* = 0.017), and Fjord horses and Welsh ponies (*p* = 0.031) differed significantly in their EHM proportions. Other comparisons did not reach statistical significance. 

We reassigned 502 animals to 6 clusters with similar genetical background (Table 1). For each cluster we determined the proportion of EHM (Figure 2). As we allocated several ‘tall horse’ breeds to cluster 1, the group size totaled 350; however, it did not alter the proportion of EHM-affected animals (30%) (compare Figure 1 with Figure 2). Welsh ponies assigned to cluster 5 were combined with few other breeds, which increased the group size in cluster 5 slightly, but more so, the EHM proportion increased to 7% within the cluster. As we combined the Icelandic horse, Shetland pony and Fjord horses in cluster 6, the group size increased to n = 68, but it also increased the EHM proportion in this cluster. Applying identical statistical methods, a pairwise Fisher’s exact test followed by Holm adjustment for multiple comparisons, differences in EHM proportions between cluster 1 and cluster 5 were reaching significance only in the uncorrected Fisher’s exact test (*p* = 0.005). We noticed the same trend between cluster 5 and cluster 6 (*p* = 0.051). With Holm adjustment for multiple comparisons, only cluster 1 and 5 differences approached statistical significance (*p* = 0.078). 

### 3.4. Vaccination 

For the next analysis, we only included animals with a documented EHV-1 vaccination history according to equine passport information. Cluster 1 was more likely vaccinated compared to the other clusters. To investigate a potential effect of vaccination on EHM frequencies, we sorted EHV-1 vaccinated, unvaccinated, and EHM frequencies for cluster 1 vs. all other (*not*-cluster 1) animals (Figure 3A,B). A total of 287 animals were included with 196 horses in cluster 1 and 91 animals in all other clusters (*not*-cluster 1). For cluster 1 there were 111 horses without signs of EHM, of which 28 (25%) were vaccinated. Among cluster 1 horses, 85 were identified with EHM, of which 10 (12%) were vaccinated (Figure 3A). In the *not*-cluster 1 group (Figure 3B) there were 50 horses/ponies without EHM, of which 9 (18%) were vaccinated. Only 5 (12%) of 41 animals in *not*-cluster- 1 with EHM were vaccinated. Analyzing the influence of an EHV vaccination using a chi-square-test, the *p*-value for cluster 1 was 0.018, indicating a benefit of vaccination, but with a weak relationship (V_Cramer_ = 0.15). With a bias factor (BF) of −1.29, indicating substantial evidence for the H_1_-hypothesis, we may assume that there is a beneficial effect of vaccination reducing the probability for EHM in cluster 1 horses overall. In *not*-cluster 1 animals, the difference of EHM in vaccinated vs. unvaccinated (9 vs. 5 animals) was less pronounced and not statistically significant (*p* = 0.445), likely because of a small sample size of vaccinated horses. Data were independent from each other. Interestingly, the percentage of vaccinated animals that still developed EHM (12%) was the same in both groups.

## 4. Discussion

The results of the present study show a highly variable incidence of EHM among the investigated outbreaks, with a seasonal accumulation during (European) winter and spring. Most outbreaks were in boarding facilities, which also implies a high volume of human traffic. All premises included in this study stabled either primarily, mostly, or a large proportion of Warmblood (WB) or American Quarter Horse (AQH). Even within single or predominant breed premises with an EHV-1 outbreak, the EHM incidence varied between 2.2–78.3%, which clearly indicates the highly variable presentation of this disease, likely influenced by multiple variables of infection dynamics. We confirm, however, increasing age and female sex as risk factors for higher EHM incidence, whereas the incidence varied distinctly between breeds in mixed-breed operations. Our findings corroborate those in a previous study on EHM outbreaks in the Netherlands between 1999–2003 [9]. Whereas animals in the Dutch study were all unvaccinated against EHV-1, according to our definition, our study included an 18% proportion of confirmed (biannually) vaccinated animals. 

Limitations: we excluded an EHM outbreak because of the specific circumstances in an equine hospital [23]. Furthermore, we cannot claim completeness, since we included all EHM outbreaks within the time period investigated, as we typically followed up on voluntary reporting. 

Here, we confirm previous findings that most EHM outbreaks in the northern hemisphere occur during the cooler time of the year, although we are aware of outbreaks that started in the midst of summer. A seasonality for EHM outbreaks is consistent with alternative data sources. Pusterla et al., 2022, found that EHV-1 in nasal swabs of horses affected by fever or respiratory disease were limited to spring months, and monthly reports from the Equine Disease Communication Center, Lexington, Kentucky, or the International Collating Centre, Newmarket, UK, show a peak in EHM reporting between late fall and late spring [24]. The general belief is that extrinsic (environmental) factors likely enhance or sustain viral transmissibility. All operations included in this study were boarding or combined training and breeding facilities. This implies frequent horse movements on-and-off the premises; frequent direct and indirect contacts between animals; and a high human traffic volume on-and-off and within the premises, which all can facilitate viral spread. While all premises were characterized by an even distribution of age and sex frequencies, our results confirm a previously described increased risk for EHM in horses with increasing age and for the female sex following an infection [13]. These risk factors may relate to differences in inflammatory pathways or immunological profiles in the aged vs. young, or to variation in stress-related or male/female reproductive endocrinology [6]. 

With only four exceptions, most operations in our study were single- or predominate- breed operations composed of WB or AQH. A total of 66% of our overall study population were either WB horses (50%), AQH (8%), Shetland (4%) or Welsh pony (4%). These breeds are common and popular in Europe and are frequently encountered at equestrian events, used for recreational riding, or kept for companionship. EHM outbreaks on single-breed operations have been described for Thoroughbred, Standardbred, WB (and sport horse crossbreeds), AMQ/Paint, and Lipizzaner [14,18,25,26]. Denmark, Germany, and Sweden, however, are also known for large and numerous operations that exclusively house Icelandic or Arabian horses, whereas Germany and neighboring Austria boast several Tyrolean Haflinger single-breed premises. Similar to the competitive WB and AQH calendar, and similar to Thoroughbred/Standardbred racing schedules, there is an extensive competitive agenda for Icelandic, Arabian, and Haflinger horses—including multiple-day competitions, equestrian training clinics, and breeding stock shows—that require mixing and mingling as well as transportation and traveling. Yet, to our knowledge, there have been no EHM outbreaks reported from single-breed operations for any of the three breeds. Interestingly, while resilient data is unavailable, Haflinger and Icelandic breed stakeholders confirm EHV-1 abortions on single-breed operations (R. Mair, Fohlenhof Ebbs, Austria, and G. Jónsson, Icelandic Horse breeder association (IPZV), Hannover, Germany, both: personal comm.). Furthermore, a recently published investigation into EHV-1 infection prevalence among Arabian horses in Egypt evidenced abortions caused by EHV-1 with only a single EHM case during four years of surveillance [27]. These observations support that EHV-1 is also prevalent among these breeds; however, despite its presence, EHM cases are rare. In the Dutch study, Goehring et al. (2006) already noticed that EHM incidence among Arabian horses and Haflingers was low and in numbers between an increased incidence for WB and a decreased incidence for Shetland and Dartmoor ponies (listed as ‘archetypical ponies’), which served as the reference (OR = 1) for odds ratio (OR) calculations [9]. Most of our Arabian horse populations in this study were housed on a single farm (farm E, Table 2). This farm was considered a truly multiple-breed herd with roughly 140 animals. This farm also housed a 26-head herd of Fjord horses, of which 13 developed EHM during this outbreak. Different from the Dutch study, we used this group as the reference for OR calculations evaluating this individual outbreak [12]. ORs suggested a decreased EHM risk for both Shetland and Welsh ponies (*p* = 0.008 and *p* = 0.007, respectively). The ORs for Arabian horses suggested a trend for a reduced risk; however, results were not statistically significant (*p* = 0.07). As EHM incidence on farm E was similar among WB and Fjord horses, it was not surprising that there were no changes in odds when these two breeds were compared. The importance of the variability in EHM incidence among the five breeds is noteworthy, as all animals were age-matched, with an equal ratio between sexes. Furthermore, all five breeds were mingling during the daytime on an outdoor paddock, and this practice was continued during the entire outbreak. In addition, Arabian and Warmblood horses (EHM in 1/12 vs. 10/22 animals), and Fjord horses and Welsh ponies (EHM in 13/26 vs. 1/21) were housed together by nights in a closed building in group husbandry [12]. We believe these findings and the disproportionate number of outbreaks reported on single/predominant WB and AQH breed farms compared to farms with predominant Icelandic, Arabian, or Haflinger populations is strong evidence to consider breed as a risk factor in the development of EHM. These findings and the peer-reviewed literature supported an increased risk for EHM among members of cluster 1 breeds compared to other clusters. Ignoring the importance of the intrinsic and extrinsic factors of multifactorial disease during an individual farm outbreak and comparing breeds in a pairwise fashion showed statistically significant results only between the Fjord horses and the Shetland and Welsh pony group (*p* = 0.017 and *p* = 0.031, respectively). These results are similar to the previous study conducted in the Netherlands, which showed a decreased EHM incidence for Shetland pony. However, here it was of no help combining breeds with similar genetical background in clusters in an attempt to increase group sizes. Specifically, when we combined Shetland pony, Icelandic and Fjord horse in cluster six, we lost statistical power because of the high EHM incidence. Fjord horses as a group were added to an otherwise low incidence group (Shetland pony). In summary, our results indicate that susceptibility for EHM is likely complex, as it is a multifactorial disease. Including available literature sources and observations as listed above, genetic factors are likely to influence susceptibility to EHM; however, they do not segregate by breed type (or cluster), as seen in cluster 6. 

A fever in the adult horse or pony following an EHV-1 infection is typically a systemic response to viremia. It is a monophasic fever for two to seven, and under experimental conditions a first day of fever is noticed between days two to five post infection [5,6]. Absence of fever in purpose-infected (adult) animals, on the other hand, correlates with no or low-grade viremia. However, as primary respiratory tract infections occur, the animal will replicate and shed virus, and the animal will seroconvert. During most outbreaks, data collection on body temperature was performed at many time points and in most animals, but not in all. During some outbreaks, we were only able to obtain an estimate of the proportion of fever in the herd. Hence, fever ‘yes/no’ during the outbreak has mostly been the only available information to us. As viremia is a prerequisite for EHM, it is not surprising that detected fever in this study had the highest OR for EHM of all risk factors investigated. This has been also shown in the past [9]. The general belief is that proinflammatory cascades and immune profiles generated during viremia will prime the CNS vasculature to allow lymphocytic/monocytic cell extravasation and endothelial cell infection [8]. While only a proportion of viremic animals develop EHM, it may depend on magnitude and duration of viremia. However, a significant difference of undetectable viremia has been noticed among vaccinates in two separate vaccination-challenge experiments in the past [10,28]. 

As >90% seroconversion for EHV-1 is assumed in a barn unit affected by EHM, and only a proportion of animals develops fever or EHM, a subclinical infection of (almost) the entire unit has to be assumed [7]. A balance between viral qualities, infectious dose, and innate/specific immunity in the host will determine the degree of clinical signs following an infection. Transmissibility of infectious virus can be mitigated by distance and barriers, whereas specific immunity against EHV-1 originates from previous exposure and/or vaccine prophylaxis. Reliable vaccination status could be obtained from 287 animals, and only a small proportion of animals were vaccinated against EHV-1 (18.1%). A slightly higher percentage of EHV-1 vaccinated horses (19.4%, 38 of 196) was detected in cluster 1 horses, whereas the percentage of vaccinated horses was 18.7% (14/91) in any not-cluster 1 animal. More than 95% of animals were vaccinated with an inactivated product against EHV-1 (and -4), which was the predominant product on the market during the observational period. As breed, age, sex and occurrence of EHM were the only objective descriptors in this outbreak evaluation and seroconversion data were unavailable, we limited our assessment on the possible effect of vaccination on EHM to cluster 1 horses. Here, we noticed a lower percentage of EHM affected horses among the EHV-1-vaccinated (12%) over the unvaccinated (or insufficiently vaccinated) horses (25%). While this calculation does not take other risk-increasing or risk-reducing factors such as distance, exposure to infectious dose, or mitigation into account, it may indicate some level of protection from viremia and EHM in the vaccinate. However, this data also suggests that regardless of breed, age, and sex, where high infectious doses and effective transmissibility are achieved, EHM will be induced, regardless. 

This study is a retrospective analysis of 13 highly variable outbreaks with data collection in 3 European countries, where local characteristics and procedures are likely to be of major importance in the course of an outbreak. In our experience, prevention or mitigation comes through rapid identification of an index case, immediate testing of animals in the vicinity of case 1, and swift transfer of any shedding animal into isolation units. However, in addition to farm-dependent factors, our findings strengthen current thoughts on other risk factors in the development of EHM. We are aware that a study such as ours does not allow stringent hypothesis testing. However, it should stimulate hypothesis generation for future projects. In fact, our findings support the reliability of the ‘old horse model’ in EHV-1 experimental infection. This model is already in use in experiments where a high proportion of EHM-affected animals is desired, without the need for large group sizes to test vaccine or drug efficacy against EHM [5,6,29].

## Figures and Tables

**Table 1:** Breeds alphabetically listed with numbers (n) of horses per breed and genetic cluster assignment (1–6).

Breed Denomination	n	n with EHM	Cluster Assignment
American Paint Horse	1	1	1
American Quarter Horse (AQH)	46	14	1
Appaloosa	1	0	1
Standardbred	2	1	1
Thoroughbred	3	2	1
Warmblood (all studbooks) (WB)	296	87	1
F1 crossbred within cluster	1	0	1
Arabian	16	3	2
Criollo	1	0	3
Lusitano	2	1	3
Mestizio	1	0	3
Pura raza espanola (PRE)	10	1	3
Draft horse	5	0	4
Friesian	3	1	4
Haflinger	16	3	4
F1 crossbred within cluster	1	0	4
Connemara	5	1	5
New Forest	1	0	5
Welsh pony (classes A–C)	23	1	5
Fjord	32	14	6
Gotland pony	1	0	6
Huzul (Carpathian)	2	0	6
Icelandic	8	2	6
Konik	1	0	6
Shetland pony	24	1	6
**Total assigned to clusters**	**502**	133	
Duelmener	1	1	unassigned
Lewitzer	1	1	unassigned
Lipizzaner	1	0	unassigned
Pinto	1	0	unassigned
Robust pony (unknown pedigree; combined based on size, built & appearance)	20	0	unassigned
Sport pony (unknown pedigree; combined based on built & use (dressage, show jumping)	40	9	unassigned
F1 crossbreeds between different cluster	15	6	unassigned
Pedigree unknown horses	8	0	unassigned
**Total**	**589**	150	

**Table 2:** EHV-1 outbreak characteristics of included premises.

Country Denmark (DK) Germany (GE) Sweden (SE)	Farm ID	Stable Units with Infection, Out of Total Units	EHV-1 Genotype	Horses in Affected Units	Horses with Fever	Horses with Neurological Signs (EHM)	EHV-1 Vaccinated Horses		Questionnaire Return Rate ^(1)^
							Total	with EHM	
GE	A	2 of 4	D	51	46 (90%)	22 (43%)	-	-	100%
GE	B	2 of 2	D	45	30 (67%)	25 (56%)	0	0	49%
GE	C *	7 of 11	D	79	55 (70%)	8 (10%)	32	4	100% ^(2)^
GE	D	4 of 4	-	45	4 (9%)	1 (2%)	-	0	38%
GE	E **	3 of 3	-	141	est. 40%	33 (23%)	1	0	100% ^(2)^
GE	F	2 of 2	-	70	11 (16%)	2 (3%)	-	-	20%
GE	G	4 of 4	D	54	23 (43%)	9 (17%)	29	5	100%
GE	H	1 of 6	-	23	19 (83%)	18 (78%)	5	5	100%
DK	I	4 of 5	D	70	68 (97%)	7 (10%)	0	0	9%
SE	J	1 of 1	D	59	35 (59%)	20 (34%)	1	0	100%
SE	K	2 of 2	D	40	22 (55%)	4 (10%)	1	0	100%
GE	L	3 of 3	N	41	15 (37%)	1 (2%)	8	0	100%
DK	M	3 of 3	-	54	42 (78%)	12 (22%)	0	0	100%
**Min**				**23**	**9%**	**2%**			**9%**
**Max**				**141**	**97%**	**78%**			**100%**
**Mean**				**594**	**59%**	**24%**			**76%**

(*) Walter et al., 2013 [18]. (**) Klouth et al., 2021 [12]. ^(1)^ % return from affected stable units only. ^(2)^ limited information provided on most questionnaires.

**Table 3:** Results of the logistic mixed model.

	EHM		
Predictors	Odds Ratios	95% CI	*p*
(Intercept)	0.02 **	0.01–0.11	**<0.001**
Age	1.06 *	1.01–1.12	**0.032**
Sex (female)	1.94 *	1.02–3.67	**0.042**
Vaccination (yes)	1.02	0.35–2.98	0.965
Fever (yes)	13.95 **	5.75–33.81	**<0.001**
**Random Effects**			
σ^2^	3.29		
^τ^00 farm	2.62		
ICC	0.44		
N_farm_	12		
Observations	339		
Marginal R²/Conditional R²	0.249/0.582		
AIC	292.679		

* *p* < 0.05, ** *p* < 0.001.

## Data Availability

The data presented in this study are available on request from the corresponding author.

## References

[1] Lunn D., Davis-Poynter N., Flaminio M., Horohov D., Osterrieder K., Pusterla N., Townsend H. (2009). Equine herpesvirus-1 consensus statement. J. Vet. Intern. Med..

[2] Wilsterman S., Soboll-Hussey G., Lunn D., Ashton L., Callan R., Hussey S., Rao S., Goehring L. (2011). Equine herpesvirus-1 infected peripheral blood mononuclear cell subpopulations during viremia. Vet. Microbiol..

[3] Edington N., Welch H., Griffiths L. (1994). The prevalence of latent equid herpesviruses in the tissues of 40 abattoir horses. Equine Vet. J..

[4] Allen G.P., Kydd J.H., Slater J.D., Smith K.C., Wernery U., Wade J., Mumford J. (1999). Advances in understanding of the pathogenesis, epidemiology and immunological control of equine herpesvirus abortion. Equine Infectious Diseases.

[5] Maxwell L.K., Bentz B.G., Gilliam L.L., Ritchey J.W., Pusterla N., Eberle R., Holbrook T.C., McFarlane D., Rezabek G.B., Meinkoth J. (2017). Efficacy of the early administration of valacyclovir hydrochloride for the treatment of neuropathogenic equine herpesvirus type-1 infection in horses. Am. J. Vet. Res..

[6] Giessler K., Goehring L., Jacobs S., McCauley A., Esser M., Lee Y., Zarski L., Weber P., Soboll Hussey G. Use of the old horse model to identify horst factors contributing to EHM pathogenesis. Proceedings of the Conference of Research Workers in Animal Diseases.

[7] van Maanen C., van Oldruitenborgh-Oosterbaan M.S., Damen E., Derksen A. (2001). Neurological disease associated with EHV-1-infection in a riding school: Clinical and virological characteristics. Equine Vet. J..

[8] Pusterla N., Hussey G.S., Goehring L.S. (2022). Equine Herpesvirus-1 Myeloencephalopathy. Vet. Clin. N. Am. Equine Pract..

[9] Goehring L.S., van Winden S.C., Van Maanen C., van Oldruitenborgh-Oosterbaan M.M.S. (2006). Equine herpesvirus type 1-associated myeloencephalopathy in the Netherlands: A four-year retrospective study (1999–2003). J. Vet. Intern. Med..

[10] Goehring L., Wagner B., Bigbie R., Hussey S., Rao S., Morley P., Lunn D. (2010). Control of EHV-1 viremia and nasal shedding by commercial vaccines. Vaccine.

[11] Nugent J., Birch-Machin I., Smith K., Mumford J., Swann Z., Newton J., Bowden R., Allen G., Davis-Poynter N. (2006). Analysis of equid herpesvirus 1 strain variation reveals a point mutation of the DNA polymerase strongly associated with neuropathogenic versus nonneuropathogenic disease outbreaks. J. Virol..

[12] Klouth E., Zablotski Y., Goehring L.S. (2021). Apparent Breed Predilection for Equid Herpesvirus-1-Associated Myeloencephalopathy (EHM) in a Multiple-Breed Herd. Pathogens.

[13] Allen G.P. (2008). Risk factors for development of neurologic disease after experimental exposure to equine herpesvirus-1 in horses. Am. J. Vet. Res..

[14] Traub-Dargatz J.L., Pelzel-McCluskey A.M., Creekmore L., Geiser-Novotny S., Kasari T., Wiedenheft A., Bush E., Bjork K. (2013). Case–control study of a multistate equine herpesvirus myeloencephalopathy outbreak. J. Vet. Intern. Med..

[15] Schnabel C.L., Babasyan S., Rollins A., Freer H., Wimer C.L., Perkins G.A., Raza F., Osterrieder N., Wagner B. (2019). An equine herpesvirus type 1 (EHV-1) Ab4 open reading frame 2 deletion mutant provides immunity and protection from EHV-1 infection and disease. J. Virol..

[16] Henninger R.W., Reed S.M., Saville W.J., Allen G.P., Hass G.F., Kohn C.W., Sofaly C. (2007). Outbreak of neurologic disease caused by equine herpesvirus-1 at a university equestrian center. J. Vet. Intern. Med..

[17] Couroucé A., Tessier C., Pomares R., Thévenot R., Marcillaud-Pitel C., Pronost S., Legrand L., Pitel P.H. EHV-1 neurological outbreak during a show jumping competition: A clinical and epidemiological study. Proceedings of the 11th International Equine Infectious Diseases Conference.

[18] Walter J., Seeh C., Fey K., Bleul U., Osterrieder N. (2013). Clinical observations and management of a severe equine herpesvirus type 1 outbreak with abortion and encephalomyelitis. Acta Vet. Scand..

[19] Petersen J.L., Mickelson J.R., Cothran E.G., Andersson L.S., Axelsson J., Bailey E., Bannasch D., Binns M.M., Borges A.S., Brama P. (2013). Genetic diversity in the modern horse illustrated from genome-wide SNP data. PLoS ONE.

[20] Felicetti M., Lopes M., Verini-Supplizi A., da Câmara Machado A., Silvestrelli M., Mendonça D., Distl O. (2010). Genetic diversity in the Maremmano horse and its relationship with other European horse breeds. Anim. Genet..

[21] Funder D.C., Ozer D.J. (2019). Evaluating effect size in psychological research: Sense and nonsense. Adv. Methods Pract. Psychol. Sci..

[22] Jeffreys H. (1998). The Theory of Probability.

[23] Vandenberghe E., Boshuizen B., Delesalle C.J., Goehring L.S., Groome K.A., van Maanen K., de Bruijn C.M. (2021). New Insights into the Management of an EHV-1 (Equine Hospital) Outbreak. Viruses.

[24] Pusterla N., Barnum S., Young A., Mendonsa E., Lee S., Hankin S., Brittner S., Finno C.J. (2022). Molecular Monitoring of EHV-1 in Silently Infected Performance Horses through Nasal and Environmental Sample Testing. Pathogens.

[25] Friday P.A., Scarratt W.K., Elvinger F., Timoney P.J., Bonda A. (2000). Ataxia and paresis with equine herpesvirus type 1 infection in a herd of riding school horses. J. Vet. Intern. Med..

[26] Bürki F., Nowotny N., Hinaidy B., Pallan C. (1984). Die Ätiologie der Lipizzanerseuche in Piber 1983: Equine Herpesvirus 1. Wien Tierärztl Mschr.

[27] Ahdy A.M., Ahmed B.M., Elgamal M.A., Shaalan M., Farag I.M., Mahfouz E.R., Darwish H.R., Sayed-Ahmed M.Z., Shalaby M.A., El-Sanousi A.A. (2022). Detection of Equid Alphaherpesvirus 1 from Arabian Horses with different clinical presentations between 2016-2019 in Egypt. J. Equine Vet. Sci..

[28] Minke J., Fischer L., Baudu P., Guigal P., Sindle T., Mumford J., Audonnet J. (2006). Use of DNA and recombinant canarypox viral (ALVAC) vectors for equine herpes virus vaccination. Vet. Immunol. Immunopathol..

[29] Allen G., Breathnach C. (2006). Quantification by real-time PCR of the magnitude and duration of leucocyte-associated viraemia in horses infected with neuropathogenic vs. non-neuropathogenic strains of EHV-1. Equine Vet. J..

